# Inflammation and its association with oxidative stress in dogs with heart failure

**DOI:** 10.1186/s12917-021-02878-x

**Published:** 2021-04-26

**Authors:** Alenka Nemec Svete, Barbara Verk, Nina Čebulj-Kadunc, Janez Salobir, Vida Rezar, Aleksandra Domanjko Petrič

**Affiliations:** 1 Small Animal Clinic, Veterinary Faculty, University of Ljubljana, Gerbičeva 60, 1000 Ljubljana, Slovenia; 2 Institute of Preclinical Sciences, Veterinary Faculty, University of Ljubljana, Gerbičeva 60, 1000 Ljubljana, Slovenia; 3 Institute of Nutrition, Biotechnical Faculty, University of Ljubljana, Groblje 3, 1230 Domžale, Slovenia

**Keywords:** Canine congestive heart failure, C–reactive protein, Interleukin–6, Malondialdehyde, Tumour necrosis factor–alpha, White blood cell count

## Abstract

**Background:**

Inflammation and oxidative stress can contribute to the development and progression of heart failure. This study aimed to investigate the association between inflammatory and oxidative stress markers in dogs with congestive heart failure (CHF). Associations between the disease severity marker N-terminal pro-B-type natriuretic peptide (NT-proBNP) and markers of inflammation and oxidative stress were also determined.

**Results:**

Thirty-seven dogs with cardiovascular diseases (dilated cardiomyopathy, DCM (16 dogs), myxomatous mitral valve disease, MMVD (21 dogs)) and ten healthy dogs were included in this prospective study. The patients were further divided into groups with (26) and without CHF (11). We found a significantly higher serum concentration of C-reactive protein (*P* = 0.012), white blood cell (*P* = 0.001), neutrophil (*P* = 0.001) and monocyte counts (*P* = 0.001) in patients with CHF compared to control dogs. The concentration of tumor necrosis factor-alpha (TNF-α) was significantly higher in patients with CHF compared to patients without CHF (*P* = 0.030). No significant difference was found in most of the measured parameters between MMVD and DCM patients, except for glutathione peroxidase (GPX) and NT-proBNP. In patients with CHF, TNF-α correlated positively with malondialdehyde (*P* = 0.014, *r* = 0.474) and negatively with GPX (*P* = 0.026, *r* = − 0.453), and interleukin-6 correlated negatively with GPX (*P* = 0.046, *r* = − 0.412). NT-proBNP correlated positively with malondialdehyde (*P* = 0.011, *r* = 0.493). In patients without CHF none of the inflammatory and oxidative stress markers correlated significantly. Furthermore, in the group of all cardiac patients, GPX activity significantly negatively correlated with NT-proBNP (*P* = 0.050, *r* = − 0.339) and several markers of inflammation, including TNF-α (*P* = 0.010, *r* = − 0.436), interleukin-6 (*P* = 0.026, *r* = − 0.382), white blood cell (*P* = 0.032, *r* = − 0.369), neutrophil (*P* = 0.027, *r* = − 0.379) and monocyte counts (*P* = 0.024, *r* = − 0.386).

**Conclusion:**

Inflammatory and oxidative stress markers are linked in canine CHF patients, but not in patients without CHF. These results suggest complex cross communication between the two biological pathways in advanced stages of CHF.

**Supplementary Information:**

The online version contains supplementary material available at 10.1186/s12917-021-02878-x.

## Background

Increasing evidence suggests that processes of inflammation are implicated in the pathophysiology and progression of congestive heart failure (CHF) [1, 2]. Inflammation can cause myocardial dysfunction, which leads to endothelial dysfunction and cardiac cachexia. Inflammatory markers are released from the cells of failing myocardium and endothelial cells, blood leukocytes, and platelets, as well as from the liver and lungs [1–3]. Markers of inflammation, such as C–reactive protein (CRP), cytokines, and their corresponding soluble receptors, white blood cell count (WBC) and markers of an activated immune system, have been reported to be elevated in human heart failure patients [4–7]. In canine patients with CHF, WBC, and concentrations of CRP and monocyte chemoattractant protein-1 have been found to be significantly higher in comparison to control dogs [8–15]. Besides, interleukin (IL)-2, IL-7, and IL-8 decreased with increasing severity indices of myxomatous mitral valve disease (MMVD) [14]. On the other hand, Rubio and colleagues found no significant differences in a number of cytokines (IL-2, IL-6, IL-7, IL-8, IL-10, IL-15, IL-18, interferon gamma-induced protein, monocyte chemoattractant protein-1, granulocyte macrophage-colony stimulating factor, tumour necrosis factor-α (TNF-α) and interferon-gamma) between dogs with different stages of heart failure due to MMVD or dilated cardiomyopathy (DCM) [15].

Inflammation and oxidative stress play an important role in various features of cardiovascular disease, involving endothelial dysfunction, lipid disorders, and myocardial injury [1, 2, 16]. Clinical and experimental studies have provided considerable evidence that oxidative stress, defined as a disturbance in the balance between reactive oxygen species (ROS) and antioxidants, is increased in heart failure and consequently contributes to cardiac remodelling and heart failure [1, 17]. Increased production of ROS and thus increased oxidative stress is implicated in the development of a variety of cardiovascular diseases in human and animal patients [1, 15, 17–22].

Congestive heart failure, regardless of etiology, is linked with inflammation and enhanced oxidative stress, whereas short-term inotropic support results in the reduction of inflammatory and oxidative stress markers [23]. Biomarkers of oxidative stress and inflammation are promising diagnostic and prognostic markers in heart failure patients [15, 20]. Despite the fact that oxidative stress and inflammation are involved in the progression of heart failure [1, 15, 24] there is still a lack of data on the association between inflammatory and oxidative stress markers in human and canine cardiovascular patients.

Our hypothesis was that inflammatory and oxidative stress markers are associated in canine patients with CHF resulting from MMVD or DCM. To test the hypothesis, we measured selected inflammatory and oxidative stress markers. We correlated them with each other, as well as with the disease severity marker N-terminal pro–B–type natriuretic peptide (NT–proBNP).

## Results

### Dogs

**Fig. 1:**
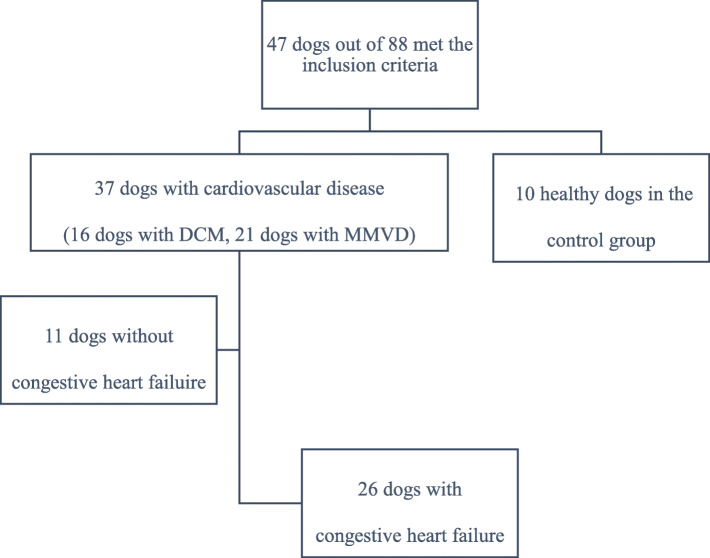
The progression algorithm of dogs during the inclusion into the study. DCM: dilated cardiomyopathy; MMVD: myxomatous mitral valve disease

Forty-seven privately owned dogs were included in the study: 37 of them were cardiovascular patients, and ten of them were control dogs. Cardiovascular patients were further divided into two groups; patients with (26) and without (11) CHF. The progression algorithm of dogs during the inclusion into the study is shown in Fig. 1. The baseline demographic characteristics of cardiovascular patients with and without CHF and control dogs are presented in Table 1. Control dogs and cardiac patients with and without CHF did not differ significantly in weight; however, patients with and without CHF were significantly (*P* < 0.001) older compared to control dogs. Furthemore, patients with and without CHF did not differ significantly in age.

**Table 1:** Baseline demographic characteristics of canine cardiac patients and control dogs

	Control	All patients	No CHF	CHF
Number	10	37	11	26
Sex				
Male/Female	3/7	31/6	9/2	22/4
Age (years)				
Mean ± SD	4.4 ± 2.5*	8.8 ± 2.8	9.3 ± 3.6	8.6 ± 2.5
Min–Max	1.0–12.5	2.4–14.3	2.4–14.3	4.2–12.5
Weight				
Median (IQR)	22.4 (19.2–34.3)	27.8 (13.2–41.9)	31.4 (13.6–61.5)	27.2 (12.8–39.3)
Disease				
DCM/MMVD	/	16/21	4/7	12/14

*significant difference when compared to the groups of canine patients with CHF (*P* < 0.001) and without CHF (*P* < 0.001), and the group of all cardiac patients (*P* < 0.001)

Age and weight (Table S1) were compared between DCM (16) and MMVD (21) patients and control dogs. Dogs with MMVD were significantly older compared to DCM patients (*P* = 0.027) and control dogs (*P* < 0.001). In addition, dogs with DCM were significantly older compared to control dogs (*P* = 0.011) and had significantly higher (*P* < 0.001) weight than MMVD dogs.

### Inflammatory and markers of oxidative stress and NT–proBNP concentrations

Concentrations of IL-6 were excluded from the statistical comparison because they were below the lower level of detection.

The results of inflammatory markers and NT–proBNP are presented in Table 2. NT–proBNP concentration was significantly higher in patients with CHF than in patients without CHF (*P* = 0.009) and control dogs (*P* < 0.001); however, NT–proBNP concentration between patients without CHF and control dogs did not differ significantly.

**Table 2:** Inflammatory markers (Median, IQR) and NT–proBNP concentrations (Median, IQR) in patients and control dogs

	Control (*n* = 10)	No CHF (*n* = 11)	CHF (*n* = 26)
TNF-α (pg/mL)	3.90; 3.90–10.50	3.90; 3.90–3.90	3.90; 6.85–11.90^a^
IL-6 (pg/mL)	31.3; 31.3–31.3	31.3; 31.3–31.3	31.3; 31.3–31.3
CRP (mg/L)	0.90; 0.78–1.33	1.45; 0.57–7.74	3.53; 1.10–13.18^b^
WBC (× 10^9^/L)	6.2; 5.2–8.2	7.7; 6.3–8.9	10.5; 8.1–13.6^c^
NEUT (× 10^9^/L)	3.4; 2.8–5.2	5.2; 3.2–6.8	7.2; 5.7–10.3^b^
Neutrophils (%)	57.3; 51.2–60.7^d^	66.3; 63.2–75.9	69.5; 61.3–73.5
LYMPH (× 10^9^/L)	1.83; 1.47–2.35	1.28; 1.18–2.26	1.96; 1.57–2.89^a^
Lymphocytes (%)	30.7; 24.8–35.0^d^	17.4; 14.2–26.0	20.0; 14.7–24.6
MONO (× 10^9^/L)	0.28; 0.21–0.39	0.42; 0.28–0.55	0.60; 0.44–0.84^c^
Monocytes (%)	4.2; 3.6–6.4	5.5; 4.5–6.2	5.7; 4.3–7.7
NT–proBNP (pmol/L)	822; 507–1201	1207; 927–3086	4773; 2828–8529^c^

^a^Significant difference (*P* < 0.05) when compared to patients without CHF; ^b^Significant difference (*P* < 0.05) when compared to control dogs; ^c^Significant difference (*P* < 0.05) when compared to control dogs and patients without CHF; ^d^Significant difference (*P* < 0.05) when compared to patients without and with CHF

Serum CRP concentration was significantly higher in patients with CHF than in control dogs (*P* = 0.012). The concentration of TNF-α was significantly (*P* = 0.030) higher in patients with CHF compared to patients without CHF, while no significant difference was found in comparison to control dogs.

White blood cell and monocyte counts were significantly higher in patients with CHF compared to control dogs (*P* = 0.001; and *P* = 0.001, respectively) and patients without CHF (*P* = 0.024 and *P* = 0.049, respectively). Neutrophil counts were significantly (*P* = 0.001) higher in patients with CHF than in control dogs. We found significantly (*P* = 0.020) higher lymphocyte counts in patients with CHF than in patients without CHF. Furthermore, we found significantly higher neutrophil percentages and significantly lower lymphocyte percentages in patients without (*P* = 0.002 and *P* = 0.010, respectively) and with CHF (*P* = 0.003 and *P* = 0.006, respectively) in comparison to control dogs.

Regarding oxidative stress parameters (Table 3), we found no significant difference in malondialdehyde (MDA) concentration between groups of patients and control dogs. On the other side, glutathione peroxidase (GPX) activity was significantly (*P* = 0.042) higher in patients without CHF than in control dogs.

**Table 3:** Oxidative stress markers (mean ± SD) in patients and control dogs

	Control *n* = 10	No CHF *n* = 11	CHF *n* = 26
MDA (μmol/L)	1.34 ± 0.31	1.77 ± 0.75	1.32 ± 0.72
GPX (U/g Hgb)	393.7 ± 43.6	457.6 ± 43.7*	429.2 ± 65.6

* Significant difference (*P* < 0.05) when compared to control dogs

Inflammatory and oxidative stress markers and NT-proBNP concentrations were also compared between DCM and MMVD patients and control dogs (Tables S2 and S3). We found no significant difference between DCM and MMVD patients in TNF-α and CRP concentrations, WBC, NEUT, LYMPH, MONO and percentages of neutrophils, lymphocytes and monocytes, and MDA concentration; however, GPX activity was significantly higher in MMVD than in DCM patients (*P* = 0.003) and control dogs (*P* = 0.003). NT-proBNP concentration was significantly higher in DCM compared to MMVD patients (*P* = 0.018) and control dogs (*P* < 0.001), while NT-proBNP in MMVD patients was significantly higher than in control dogs (*P* = 0.010). On the other hand, we found a significant difference (*P* < 0.05) between the control dogs and both groups of patients (MMVD and DCM) in WBC, NEUT, MONO and percentages of neutrophils and lymphocytes.

### Correlations

In CHF patients, TNF-α concentrations correlated significantly positively with MDA concentrations (*P* = 0.014, *r* = 0.474) and negatively with GPX activities (*P* = 0.026, *r* = − 0.453). Furthermore, we found a significant negative correlation (*P* = 0.046, *r* = − 0.412) between IL-6 concentrations and GPX activities. Percentages of neutrophils and monocyte counts correlated significantly negatively with GPX activities (*P* = 0.024, *r* = − 0.460 and *P* = 0.031, *r* = − 0.441, respectively). On the other hand, percentage of lymphocytes correlated significantly positively with GPX activities (*P* = 0.006, *r* = 0.542), and lymphocyte counts correlated significantly negatively with MDA concentrations (*P* = 0.050, *r* = − 0.388). In this group of patients, we found a significant positive correlation between NT-proBNP and MDA concentrations (*P* = 0.011, *r* = 0.493).

In patients without CHF, none of the inflammatory and oxidative stress markers correlated significantly.

Additionally, we correlated inflammatory and oxidative stress markers and NT-proBNP in the group of all 37 patients and found several significant correlations. Interestingly, GPX activities correlated significantly negatively with several inflammatory markers, including TNF-α concentrations (*P* = 0.010, *r* = − 0.436), IL-6 concentrations (*P* = 0.026, *r* = − 0.382), WBC (*P* = 0.032, *r* = − 0.369), and neutrophil (*P* = 0.027, *r* = − 0.379) and monocyte counts (*P* = 0.024, *r* = − 0.386). On the other hand, GPX activities correlated significantly positively with percentages of lymphocytes (*P* = 0.043, *r* = 0.348). Furthermore, we found a significant negative correlation (*P* = 0.009, *r* = − 0.423) between MDA concentrations and lymphocyte counts. In the group of all pateints, NT-proBNP concentrations correlated significantly negatively with GPX activities (*P* = 0.050, *r* = − 0.339) and positively with TNF-α concentrations (*P* = 0.022; *r* = 0.378) and monocyte count (*P* = 0.019, *r* = 0.385).

## Discussion

This paper reports novelties with respect to markers of inflammation and oxidative stress in canine cardiac patients and their relationship as well as their correlation with the disease severity marker NT–proBNP. Numerous papers reported that heart failure is characterized by local and systemic chronic inflammation [1, 2]. Studies in people have already demonstrated that oxidative stress and chronic inflammation are linked in heart failure patients [1, 2, 16, 20, 25, 26]. Canine and human patients with heart failure have elevated levels of a number of inflammatory markers [1, 8, 10, 12–15, 20]. Proinflammatory cytokines, especially TNF-α, IL-1, and IL-6, aggravate hemodynamic abnormalities, exert direct toxic effects on the heart and play a role in inflammation, tissue wasting, and weight loss [2]. It has been reported that dogs with CHF had alerations in blood mRNA expression of inflammatory and pro- and anti-fibrotic markers. Namely, dogs with decompensated CHF had significantly higher mRNA levels of the proinflammatory cytokines (IL1-β and IL-2), matrix metalloproteinase1, and tissue inhibitor of metalloproteinase (TIMP3) and lower TNF-α, transforming growth factorβ3, TIMP1 and TIMP2 compared to healthy dogs [27]. In addition, altered myocardial cytokine expression was demonstrated in dogs with systemic and end-stage cardiac disease compared with control dogs, and dogs with end-stage cardiac disease had significantly increased myocardial expression of interleukins (IL -1, IL -6, IL -8, IL -10), TNF-α, and interferon-gamma compared with dogs with systemic disease [28]. Our results showed significantly higher TNF-α concentration in patients with CHF compared to patients without CHF, but not compared to healthy dogs. Significantly higher TNF concentration was determined in dogs with CHF due to DCM compared to healthy dogs in the preliminary study of Freeman and colleagues [29]. However, different methodology was used in our study (ELISA, TNF-α) and theirs (cytotoxicity bioassay, TNF) to determine the concentration of this cytokine. In another study, Freeman and colleagues found no significant difference in TNF concentration between healthy dogs and canine patients with CHF due to DCM, which is in line with our results [30]. In the studies of Zois and colleagues [14] and Kim and colleagues [31] TNF-α was not quantifiable in any MMVD dog. Furthermore, no significant difference in TNF-α concentration was observed in dogs with different stages of heart failure due to MMVD or DCM [15]. Due to negative inotropism of TNF–α, this cytokine contributes to interstitial fibrosis, myocyte apoptosis, ventricular remodelling, and systolic dysfunction, [27]. Several papers demonstrated the increased circulatory concentration of proinflammatory cytokines in human heart failure patients [4, 25, 32]. In our study majority of dogs (87%) had non-quantifiable concentrations of IL-6, which is in agreement with the study of Zois and colleagues where more than 75% of dogs had non-quantifiable concentrations of this cytokine [14].

C-reactive protein, WBC, and white blood cell differential counts are common markers of systemic inflammation. In our study serum CRP concentration was significantly higher in CHF patients in comparison to healthy dogs. Increased levels of CRP in dogs with various cardiovascular diseases and CHF have also been found in other studies [9, 11, 12, 15, 33]. In our study, significantly higher WBC and monocyte counts and neutrophil percentages and counts compared to control dogs and/or non-CHF dogs and significantly increased neutrophil percentages in patients without CHF compared to control dogs indicate some degree of inflammation. Although the median values of WBC and white blood cell differential counts were within our reference ranges, significant differences in these parameters between our cardiac patients and healthy dogs indicate the development of the inflammatory process. The results are partially in accordance with other studies [8, 10, 13, 15] and our previous study [12]. There have been a few studies investigating WBC and white blood cell differential counts in people [7, 34, 35]. Wall stress present in overloaded heart, causes sterile inflammation that contributes to heart failure. Ischemia in various tissues, including skeletal muscles and gut as a consequence of vasoconstriction and underperfusion in heart failure, also induces inflammation. Underperfused intestinal mucosa leads to increased gut permeability and enhances the translocation of bacteria and their toxins into the blood, thus contributing to systemic inflammation [36].

Increased production of ROS, induced by various triggers, is associated with the severity of inflammation. Decreased pulmonary capillary clearance of ROS positive WBC and platelets in CHF as a consequence of pulmonary congestion increases the production of ROS by mitochondria. A significantly higher number of ROS positive WBC and platelets were found in human patients with CHF in comparison to controls. It has been suggested that circulating WBC, and platelets boost oxidative stress in CHF [37]. Our oxidative stress parameters included MDA, a marker of lipid peroxidation, and GPX, one of the three major intracellular antioxidant enzymes. We found no significant difference in MDA concentrations between groups of cardiac patients and control dogs; however, the activity of GPX was significantly higher in patients without CHF than in control dogs. Similar results were found in canine cardiovascular patients by Freeman and colleagues [21, 22]. These investigators reported no significant difference in MDA concentration between CHF patients and healthy dogs while they found a significantly higher concentration of another reliable biomarker of lipid peroxidation, 8-F_2α_-isoprostanes, in the CHF patients compared to healthy dogs [22]. According to our results regarding MDA concentrations, we could assume that oxidative damage to lipids less likely occurred in our cardiac patients; however, that should be further investigated by determination of other lipid peroxidation biomarkers. Increased GPX activity in patients without CHF may indicate a compensatory response to increased production of ROS in the early phase of heart disease. In human CHF patients, increased concentration of MDA [19, 38] and decreased GPX activity have been reported [19, 39].

We found many significant correlations that indicate an association between the inflammatory process and oxidative stress in our CHF patients but not in patients without CHF. The significant positive correlation between TNF-α and MDA found in our study indicates that there is a higher degree of lipid peroxidation with an increased concentration of TNF-α. In human patients with CHF lipid peroxidation markers (MDA, lipid peroxide), and soluble receptors of TNF-α correlated significantly positively [19]. Our results are in agreement with the results of Tsutamoto and colleagues [25], who found a relationship between TNF-α and oxidative stress in patients with dilated cardiomyopathy. No significant correlations between TNF-α and total antioxidant capacity and total thiol were found in canine cardiovascular patients with DCM and MMVD [15]. TNF-α induces ROS synthesis via endothelial mitochondria and NAD(P)H and the plasma membrane; furthermore, TNF-α is implicated in the regulation of nitric oxide metabolism [32]. Negative correlations of proinflammatory cytokines TNF-α and IL-6 with GPX suggest that GPX activity decreases with increased inflammation in our CHF patients. This statement is also supported by the negative correlations of neutrophil and monocyte counts with GPX activity. Conversely, percentages of lymphocytes significantly positively correlated with GPX, and lymphocyte count significantly negatively correlated with MDA. The obtained correlations further confirm that inflammation and oxidative stress are associated in canine patients with CHF.

In our study, markers of inflammation and oxidative stress and NT-proBNP correlated significantly in the group of all cardiac patients. Interestingly, GPX activity significantly negatively correlated with several markers of inflammation, such as TNF-α, IL-6, WBC, and neutrophil and monocyte counts. These results suggest the importance of this antioxidant enzyme in cross communication between inflammatory processes and oxidative stress in canine cardiac patients. Furthermore, GPX significantly positively correlated with the percentage of lymphocytes, whereas MDA concentration significantly negatively correlated with lymphocyte count. Additionally, in the group of all patients, we found a significant negative correlation of NT-proBNP with GPX and a significant positive correlation with TNF-α and monocyte count. These results suggest that the severity of the disease is associated with a decrease in GPX activity and increased inflammation. All these correlations further document the cross communication between inflammation and oxidative stress in canine cardiac patients. Similarly, Rubio and colleagues found significant correlations between selected inflammatory parameters and antioxidant biomarkers and echocardiographic variables, suggesting that inflammation and oxidative stress collaborate in the pathogenesis of heart failure [15].

Although, it was not our major goal, we compared oxidative and inflammatory parameters between DCM and MMVD patients and healthy dogs. Interestingly, we found no significant differences in any of the inflammatory parameters between DCM and MMVD patients, but there was a significant difference in several inflammatory parameters between healthy and both groups of patients (supplementary material) indicating ongoing inflammation in both diseases. Similarly, Hamilton-Elliott and colleagues found no significant differences in individual white blood cell differentials between DCM and MMVD patients [13]. In contrast to our results, the study by de Laforcade and colleagues reported significantly lower TNF levels in DCM compared with MMVD [40]; the difference could be due to methodological reasons. Malondialdehyde concentration did not differ significantly between all investigated groups (DCM and MMVD patients, healthy dogs), which suggests that the extent of lipid peroxidation process is not affected by disease itself. Similar results were found by Freeman and colleagues who reported no significant difference in both lipid peroxidation markers, MDA and 8-F_2α_-isoprostanes [22]. Contrary to MDA, we found a significantly higher GPX activity in MMVD dogs in comparison to DCM and control dogs. This might be due to the effect of age, as the MMVD patients were significantly older than the DCM patients and control dogs, or disease etiology. Significanlty higher GPX activity was found in older healthy male dogs comparing to young males [41]. In our study males predominated in both diseases. A significant increase of whole blood GPX with aging was reported in Labrador Retrievers [42]. A recent study in healthy dogs reported a tendency of higher GPX activity in old dogs [43]. As expected, NT-proBNP concentration was significantly higher in DCM and MMVD patients in comparison to healthy dogs; at the same time DCM patients had significantly higher NT-proBNP than MMVD patients. The latter might be due to the fact that more DCM than MMVD dogs were in CHF.

Our study has some limitations that should be noted. Sex and age were not matched between our patients and control dogs. Age and/or sex, as well as neuter status, might influence the measured parameters [44, 45].

## Conclusion

In conclusion, most of the measured inflammatory markers were significantly higher in canine patients with CHF than in control dogs. Furthermore, we found that the activity of the antioxidant enzyme, GPX, decreased with increasing concentration of inflammatory markers in the group of all cardiac patients. Significant correlations between markers of inflammation and oxidative stress in patients with CHF but not in patients without CHF suggest complex cross communication between the two biological pathways with the development of CHF.

## Methods

### Dogs

In the present study, 88 privately owned dogs were evaluated. Forty-one dogs were excluded due to allergic diseases, cancer, kidney disease, endocrine disorders, or infectious diseases. Thirty-seven patients with either MMVD or DCM and ten healthy control dogs were prospectively recruited. The same cohort of dogs have been used in our previous study [46]. The ten control dogs were considered healthy according to their history, physical examination, and the routine laboratory results (haematology, biochemical profiles), as well as NT-proBNP levels. Myxomatous mitral valve disease, DCM and heart failure were diagnosed with the help of history, clinical examination, standard electrocardiogram, thoracic radiography, and echocardiography (Vingmed System Five, General Electric Healthcare, Milwaukee, Wisconsin, USA) with one- and two-dimensional modes and colour and spectral Doppler modes. Diseases were diagnosed following ECVIM/ACVIM guidelines [47]. Patients were classified as subclinical (without signs of congestive heart failure) DCM and MMVD or congestive heart failure groups. Congestive heart failure was diagnosed on the basis of clinical signs and the presence of pulmonary oedema on radiographs and supported by signs of increased pressure in the left atrium with the use of echocardiography. Cardiac therapy included diuretics, angiotensin-converting enzyme (ACE) inhibitors, pimobendan, beta blockers, and digoxin, depending on the needs of individual patients.

Signed consent was granted by the owners of the dogs. The Ethical Committee of the Ministry of Agriculture, Forestry and Food, Veterinary Administration of the Republic of Slovenia (Animal Protection Act UL RS 43/2007) approved all procedures.

### Blood sample collection and processing

Blood samples for all laboratory analyses were collected from fasted dogs. Samples for determination of haematological parameters were analysed within one hour after collection. Serum tubes (Vaccuette; Greiner Bio-One, Kremsmünster, Austria) stood for 30 min at room temperature before centrifugation for 10 min at 1300×g at room temperature. The serum used for CRP, TNF-α, and IL-6 measurements was frozen at − 80 °C and analysed in batch at the end of the study. Serum biochemical parameters were measured on the day of collection. EDTA tubes (Vacuette; Greiner Bio-One, Kremsmunster, Austria) were used for the collection of samples for measurement of plasma NT-proBNP and MDA concentrations. These tubes were centrifuged for 15 min at 1500×g at 4 °C and obtained plasma samples immediately frozen at − 80 °C until analysis. Concentrations of NT-proBNP and MDA were measured in batch at the end of the study. Blood samples for determination of whole blood GPX activity were collected into tubes containing the anticoagulant lithium heparin (Vacuette; Greiner Bio-One, Kremsmunster, Austria). Aliquots of heparinized whole blood were prepared and immediately frozen at − 80 °C until analysed in batch.

### Haematological and biochemical analyses

An automated haematology analyser ADVIA 120 (Siemens, Munich, Germany) was used for haematological measurements. Biochemical parameters (results not reported; urea, creatinine, alkaline phosphatase, alanine aminotransferase;) were measured using automated biochemistry analyser RX Daytona (Randox, Crumlin, United Kingdom). The concentrations of sodium, potassium, and chloride were measured using an electrolyte analyser ILyte (Instrumentation Laboratory, Lexington, MA, USA).

### Determination of CRP, TNF–α and IL–6 concentrations

Serum CRP, TNF-α, and IL-6 concentrations were measured with commercially available canine-specific ELISA kits (Canine CRP ELISA; Alpco, Salem, NH, USA; Canine TNF-alpha Quantikine ELISA kit; R&D Systems, Minneapolis, MN, USA; Canine IL − 6 Quantikine ELISA kit; R&D Systems, Minneapolis, MN, USA) based on quantitative sandwich enzyme immunoassay procedures. Assays were performed according to the original manufacturer’s instructions with all samples assayed in duplicate. The concentrations of TNF-α and IL-6 in serum were measured directly, whereas a sample dilution of 1:1000 was used for the measurement of CRP, which was taken into account in the calculation of the final results. The ranges of the tests were 3.9–250 pg/mL (sensitivity 2.4 pg/mL) for TNF- α, 31.3–1000 pg/ml (sensitivity 6.1 pg/mL) for IL-6 and 3.1–200 ng/mL (sensitivity 3.1 ng/mL) for CRP. The precision of the assays was monitored using control sera included in the kits with known concentrations of the analytes. Coefficients of variation were 8.9% for TNF- α, 2.75% for IL-6, and 9.8% for CRP.

### Determination of GPX activity in whole blood

Glutathione peroxidase activity was measured spectrophotometrically with an automated biochemistry analyser RX Daytona (Randox, Crumlin, Great Britain) using a commercial Ransel kit (Randox, Crumlin, Great Britain), which is based on the method of Paglia and Valentine [48]. According to the method, GPX activity is determined indirectly by measuring the rate of formation of oxidized glutathione. GPX activity was expressed as units per gram of haemoglobin (U/g Hgb). Haemoglobin concentration in the whole blood haemolysates was determined spectrophotometrically by the cyano-methaemoglobin method using an automated biochemistry analyser RX Daytona (Randox, Crumlin, Great Britain).

### Determination of MDA and NT-proBNP concentrations

The plasma MDA concentration was analysed according to a method described elsewhere [46, 49]. Briefly, two hundred μL of plasma was added to a 2-mL plastic microcentrifuge tube. The sample was mixed with 20 μL 0.2% butylated hydroxytoluene (BHT) and 200 μL 0.44 M H_3_PO_4_ and allowed to stand for 15 min. Absolute ethanol (600 μL) was added to each sample, and the samples were centrifuged (15,000×*g*, 15 min, 4 °C). In the glass tubes, the supernatant (700 μL) was mixed with 1.5 mL of 0.44 M H_3_PO_4_, 1.5 mL of 0.6% thiobarbituric acid (TBA), and 0.3 mL of ultrapure water and heated at 90 °C for 60 min. The MDA-TBA_2_ adduct was separated using an Agilent HPLC system (Santa Clara, CA, USA) equipped with a 1260 Infinity FLD fluorescence detector. The mobile phase consisted of 50 mmol/l of a 65% KH_2_PO_4_ buffer (pH 6.9) and 35% methanol. The flow rate of the mobile phase was 1.0 mL/min. A 10-μL aliquot was injected into a reversed-phase HPLC column (HyperClone 5-μm octadecylsilane [C18] 1, 4.6 mm × 150 mm × 5 μm, Phenomenex Inc., Torrance, CA, USA) and C18 octadecylsilane guard column (4 × 3 mm, Phenomenex Inc.). The results of the analysis were evaluated using OpenLab CSD Chem Station ed. rev. C.01.05 (35) software.

All frozen EDTA plasma samples were sent on dry ice to IDEXX laboratory (IDEXX Laboratories Inc., Maine, USA) by air transport. Plasma NT–proBNP concentrations were measured in IDEXX laboratory using a second-generation Cardiopet® proBNP enzyme-linked immunosorbent assay (ELISA; IDEXX Laboratories Inc., Westbrook, Maine, USA). The second-generation canine NT-proBNP assay is a sandwich ELISA with colorimetric end-point detection at 450 nm for quantitative determination of canine NT-proBNP. The second-generation assay eliminates the need for the specialized protease inhibitor blood collection tube by using new antibodies that target a more stable contiguous fragment of NT-proBNP and offers an upper reporting limit of 10000 pmol/L [50].

### Statistical analysis

Commercial software (IBM® SPSS® 24, Chicago, IL, USA) was used for data analysis. The normality test (Shapiro-Wilk) was used to assess the distribution of the data. Parameters of interest were compared between groups of dogs (cardiac patients with or without CHF and healthy dogs; DCM and MMVD and healthy dogs) using one-way ANOVA and Tukey’s HSD test (Gaussian distribution of the data), or Kruskal-Wallis test followed by Bonferroni’s method for multiple pairwise comparisons (non-Gaussian distribution of the data). An independent t-test (Gaussian distribution of the data) and the Mann-Whitney U test (non-Gaussian distribution of the data) were used to compare age and weight between healthy dogs and all patients, respectively. The results are reported as means and standard deviations (SD) (Gaussian distribution of the data) and as a median and interquartile range (IQR, 25th to 75th percentile; non-Gaussian distribution of the data). In the groups of patients with and without CHF and the group of all cardiac patients, we performed correlation analyses (non-parametric Spearman’s rank correlation) to assess associations between inflammatory and oxidative stress markers and between NT-proBNP and markers of inflammation and oxidative stress. A probability value of less than 0.05 was considered statistically significant.

## Supplementary Information

**Additional file 1: Table S1.** Baseline demographic characteristics of canine DCM and MMVD patients and control dogs. **Table S2.** Inflammatory markers (Median, IQR) and NT-proBNP concentrations (Median, IQR) in DCM and MMVD patients and control dogs. **Table S3.** Oxidative stress markers (mean ± SD) in DCM and MMVD patients and control dogs.

## Data Availability

The datasets used and/or analysed during the current study are available from the corresponding author on reasonable request.

## Abbreviations

CHF: Congestive heart failure
CRP: C-reactive protein
DCM: Dilated cardiomyopathy
GPX: Glutathione peroxidase
IL: Interleukin
IQR: Interquartile range
LYMPH: Lymphocyte count
MDA: Malondialdehyde
MMVD: Myxomatous mitral valve disease
MONO: Monocyte count
n: Number of dogs
NEUT: Neutrophil count
NT-proBNP: N-terminal pro-B-type natriuretic peptide
ROS: Reactive oxygen species
TNF-α: Tumor necrosis factor-alpha
SD: Standard deviation
WBC: White blood cell count

## References

[1] Aimo A, Castiglione V, Borrelli C, Saccaro LF, Franzini M, Masi S (2020). Oxidative stress and inflammation in the evolution of heart failure: from pathophysiology to therapeutic strategies. Eur J Prev Cardiol.

[2] Yndestad A, Damås JK, Oie E, Ueland T, Gullestad L, Aukrust P (2006). Systemic inflammation in heart failure – the whys and wherefores. Heart Fail Rev.

[3] Briasoulis A, Androulakis E, Christophides T, Tousoulis D (2016). The role of inflammation and cell death in the pathogenesis, progression and treatment of heart failure. Heart Fail Rev.

[4] Liu M, Chen J, Huang D, Ke J, Wu W (2014). A meta-analysis of proinflammatory cytokines in chronic heart failure. Heart Asia.

[5] Pellicori P, Zhang J, Cuthbert J, Urbinati A, Shah P, Kazmi S (2020). High-sensitivity C-reactive protein in chronic heart failure: patient characteristics, phenotypes, and mode of death. Cardiovasc Res.

[6] Petretta M, Condorelli GL, Spinelli L, Scopacasa F, de Caterina M, Leosco D (2000). Circulating levels of cytokines and their site of production in patients with mild to severe chronic heart failure. Am Heart J.

[7] Pfister R, Sharp SJ, Luben R, Wareham NJ, Khaw KT (2012). Differential white blood cell count and incident heart failure in men and women in the EPIC-Norfolk study. Eur Heart J.

[8] Farabaugh AE, Freeman LM, Rush JE, George KL (2004). Lymphocyte subpopulations and hematologic variables in dogs with congestive heart failure. J Vet Intern Med.

[9] Cunningham SM, Rush JE, Freeman LM (2012). Systemic inflammation and endothelial dysfunction in dogs with congestive heart failure. J Vet Intern Med.

[10] Deepti BR, Yathiraj S (2015). Hematological and biochemical variables in congestive heart failure in dogs. Int J Sci Environ Technol.

[11] Reimann MJ, Ljungvall I, Hillström A, Moller JE, Hagman R, Falk T (2016). Increased serum C–reactive protein concentrations in dogs with congestive heart failure due to myxomatous mitral valve disease. Vet J.

[12] Domanjko Petrič A, Lukman T, Verk B, Nemec SA (2018). Systemic inflammation in dogs with advanced-stage heart failure. Acta Vet Scand.

[13] Hamilton-Elliot J, Ambrose E, Christley R, Dukes-McEwan J (2018). White blood cell differentials in dogs with congestive heart failure (CHF) in comparison to those in dogs without cardiac disease. J Small Anim Pract.

[14] Zois NE, Moesgaard SG, Kjelgaard-Hansen M, Rasmussen SE, Falk T, Fossing C (2012). Circulating cytokine concentrations in dogs with different degrees of myxomatous mitral valve diesease. Vet J.

[15] Rubio CP, Saril A, Kocaturk M, Tanaka R, Koch J, Ceron JJ (2020). Changes of inflammatory and oxidative stress biomarkers in dogs with different stages of heart failure. BMC Vet Res.

[16] Pashkow FJ, Watumull DG, Campbell CL (2008). Astaxanthin: a novel potential treatment for oxidative stress and inflammation in cardiovascular disease. Am J Cardiol.

[17] Tsutsui H, Kinugawa S, Matsushima S (2011). Oxidative stress and heart failure. Am J Physiol Heart Circ Physiol.

[18] McMurray J, Chopra M, Abdullah I, Smith WE, Dargie HJ (1993). Evidence of oxidative stress in chronic heart failure in humans. Eur Heart J.

[19] Keith M, Geranmayegan A, Sole MJ (1998). Increased oxidative stress in patients with congestive heart failure. J Am Coll Cardiol.

[20] Szczurek W, Szygula-Jurkiewicz B (2015). Oxidative stress and inflammatory markers – the future of heart failure diagnostics?. Kardiochir Torakochirurgia Pol.

[21] Freeman LM, Brown DJ, Rush JE (1999). Assessment of degree of oxidative stress and antioxidant concentrations in dogs with idiopathic dilated cardiomyopathy. J Am Vet Med Assoc.

[22] Freeman LM, Rush JE, Milbury PE, Blumberg JB (2005). Antioxidant status and biomarkers of oxidative stress in dogs with congestive heart failure. J Vet Intern Med.

[23] White M, Ducharme A, Ibrahim R, Whittom L, Lavoie J, Guertin MC (2006). Increased systemic inflammation and oxidative stress in patients with worsening congestive heart failure: improvement after short-term inotropic support. Clin Sci (Lond).

[24] Siti HN, Kamisaha Y, Kamisaha J (2015). The role of oxidative stress, antioxidants and vascular inflammation in cardiovascular disease (a review). Vasc Pharmacol.

[25] Tsutamoto T, Wada A, Matsumoto T, Maeda K, Mabuchi N, Hayashi M (2001). Relationship between tumor necrosis factor–alpha production and oxidative stress in the failing hearts of patients with dilated cardiomyopathy. J Am Coll Cardiol.

[26] Pashkow FJ (2011). Oxidative stress and inflammation in heart disease: do antioxidants have a role in treatment and/or prevention?. Int J Inflamm.

[27] Fonfara S, Tew SR, Cripps P, Dukes-McEwan J, Clegg PD (2012). Increased blood mRNA expression of inflammatory and anti-fibrotic markers in dogs with congestive heart failure. Res Vet Sci.

[28] Fonfara S, Hetzel U, Tew SR, Cripps P, Dukes-McEwan J, Clegg PD (2013). Myocardial cytokine expression in dogs with systemic and naturally occurring cardiac diseases. Am J Vet Res.

[29] Freeman LM, Rush JE, Brown DJ, Roubenoff R (1994). Elevated concentrations of tumor necrosis factor in dogs with congestive heart failure. J Vet Intern Med.

[30] Freeman LM, Rush JE, Kehayias JJ, Ross JN, Meydani SN, Brown DJ (1998). Nutritional alterations and the effect of fish oil supplementation in dogs with heart failure. J Vet Intern Med.

[31] Kim HS, Kang JH, Jeung EB, Yang MP (2016). Serum concentration of leptin and adiponectin in dogs with myxomatous mitral valve disease. J Vet Intern Med.

[32] Chen O, Patel J, Mohamed E, Greene M, Moskovits N, Shani J (2014). The Immunoregulatory role of cytokines in congestive heart failure. Interdiscip J Microinflam.

[33] Rush JE, Lee ND, Freeman LM, Brewer B (2006). C-reactive protein concentration in dogs with chronic valvular disease. J Vet Intern Med.

[34] Engström G, Melander O, Hedblad B (2009). Leukocyte count and incidence of hospitalizations due to heart failure. Circ Heart Fail.

[35] Ostrowska M, Ostrowski A, Łuczak M, Jaguszewski M, Adamski P, Bellwon J (2017). Basic laboratory parameters as predictors of in-hospital death in patients with acute decompensated heart failure: data from a large single-Centre cohort. Kardiol Pol.

[36] Van Linthout S, Tschöpe C (2017). Inflammation – cause or consequence of heart failure or both?. Curr Heart Fail Rep.

[37] Ijsselmuiden AJ, Musters RJ, de Ruiter G, van Heerebeek L, Alderse-Baas F, van Schilfgaarde M (2008). Circulating white blood cells and platelets amplify oxidative stress in heart failure. Nat Clin Pract Cardiovasc Med.

[38] Serdar A, Yesilbursa D, Serdar Z, Dirican M, Turel B, Cordan J (2001). Relation of functional capacity with the oxidative stress and antioxidants in chronic heart failure. Congest Heart Fail.

[39] Polidori MC, Praticó D, Savino K, Rokach J, Stahl W, Mecocci P (2004). Increased F2 isoprostane plasma levels in patients with congestive heart failure are correlated with antioxidant status and disease severity. J Card Fail.

[40] De Laforcade AM, Freeman LM, Rush JE (2003). Serum nitrate and nitrite in dogs with spontaneous cardiac disease. J Vet Intern Med.

[41] Vajdovich P, Gaal T, Szilagyi A, Harnos A (1997). Changes in some red blood cell and clinical laboratory parameters in young and old beagle dogs. Vet Res Commun.

[42] Stowe HD, Lawler DF, Kealy RD (2006). Antioxidant status of pair-fed Labrador retrievers is affected by diet restriction and aging. J Nutr.

[43] Tomsič K, Seliškar A, Lukanc B, Nemec SA (2016). Plasma total antioxidant capacity and activities of blood glutathione peroxidase and superoxide dismutase determined in healthy dogs by using commercially available kits. Acta Veterinaria.

[44] Radakovich LB, Pannone SC, Truelove MP, Olver CS, Santangelo KS (2017). Hematology and biochemistry of aging – evidence of ‘anemia of the elderly’ in old dogs. Vet Clin Pathol.

[45] Reimann MJ, Haggstrom J, Moller JE, Lykkesfeldt J, Falk T, Olsen LH (2017). Markers of oxidative stress in dogs with myxomatous mitral valve disease are influenced by sex, neuter status, and serum cholesterol concentration. J Vet Intern Med.

[46] Verk B, Nemec Svete A, Salobir J, Rezar V, Domanjko PA (2017). Markers of oxidative stress in dogs with heart failure. J Vet Diagn Investig.

[47] Dukes-McEwan J, Borgarelli M, Tidholm A, Vollmar AC, Haggstrom J (2003). Proposed guidelines for the diagnosis of canine idiopathic dilated cardiomyopathy. J Vet Cardiol.

[48] Paglia DE, Valentine WN (1967). Studies on the quantitative and qualitative characterisation of erythrocyte glutathione peroxidase. J Lab Clin Med.

[49] Rezar V, Frankič T, Levart A, Salobir J (2010). Dose-dependent effects of F2-toxin on performance, lipid peroxidation, and genotoxicity in broiler chickems. Poult Sci.

[50] Cahill RJ, Pigeon K (2015). Strong–Townsend MI, Drexel JP, Clark GH, Buch JS. Analytical validation of a second-generation immunoassay for the quantification of N-terminal pro-B-type natriuretic peptide in canine blood. J Vet Diagn Investig.

