# Supplementary material for: Inflammation and its association with oxidative stress in dogs with heart failure
Source: BMC Vet Res. 2021 Apr 26;17:176. doi: 10.1186/s12917-021-02878-x (PMC8077822; doi:10.1186/s12917-021-02878-x)
Supplement: Supplementary file 1 — Additional file 1: Table S1. Baseline demographic characteristics of canine DCM and MMVD patients and control dogs. Table S2. Inflammatory markers (Median, IQR) and NT-proBNP concentrations (Median, IQR) in DCM and MMVD patients and control dogs. Table S3. Oxidative stress markers (mean ± SD) in DCM and MMVD patients and control dogs. [file 12917_2021_2878_MOESM1_ESM.docx]

Supplementary material

**Table S1** Baseline demographic characteristics of canine DCM and MMVD patients and control dogs

|  | Control | DCM | MMVD |
| --- | --- | --- | --- |
| Number | 10 | 16 | 21 |
| Sex  Male/Female | 3/7 | 14/2 | 17/4 |
| Age (years)  Mean ± SD  Min–Max | 4.4 ± 2.5^a^  1.0–12.5 | 7.5 ± 2.5^b^  4.0–11.9 | 9.8 ± 2.7  2.4–14.3 |
| Weight  Median (IQR) | 22.4 (19.2–34.3) | 43.0 (34.9–64.9)^b^ | 13.4 (10.9–25.9) |

^a^significant difference (*P* < 0.05) when compared to the groups of canine patients with DCM and MMVD; ^b^Significant difference (*P* < 0.05) when compared to MMVD patients

**Table S2** Inflammatory markers (Median, IQR) and NT-proBNP concentrations (Median, IQR) in DCM and MMVD patients and control dogs

|  | Control  (n = 10) | DCM  (n = 16) | MMVD  (n = 21) |
| --- | --- | --- | --- |
| TNF-α (pg/mL) | 3.90; 3.90–10.50 | 6.60; 3.90–14.86 | 3.90; 3.90–6.85 |
| IL-6 (pg/mL) | 31.3; 31.3–31.3 | 31.3; 31.3–62,5 | 31.3; 31.3–31.3 |
| CRP (mg/L) | 0.90; 0.78–1.33^a^ | 7.74; 1.69–12.00 | 1.45; 0.99–5.48 |
| WBC (x 10^9^/L) | 6.2; 5.2–8.2^b^ | 10.2; 7.4–12.6 | 9.5; 6.8–12.6 |
| NEUT (x 10^9^/L) | 3.4; 2.8–5.2 ^b^ | 6.6; 4.7–9.6 | 6.6; 4.8–9.0 |
| Neutrophils (%) | 57.3; 51.2–60.7 ^b^ | 69.9; 61.8–75.7 | 66.3; 61.4–74.0 |
| LYMPH (x 10^9^/L) | 1.83; 1.47–2.35 | 1.28; 1.39–2.72 | 1.96; 1.37–2.31 |
| Lymphocytes (%) | 30.7; 24.8–35.0 ^b^ | 18.7; 13.8–24.2 | 20.1; 15.8–26.4 |
| MONO (x 10^9^/L) | 0.28; 0.21–0.39 ^b^ | 0.57; 0.43–0.95 | 0.50; 0.41–0.73 |
| Monocytes (%) | 4.2; 3.6–6.4 | 6.1; 4.9–7.1 | 4.9; 4.3–7.0 |
| NT–proBNP (pmol/L) | 822; 507–1201 ^b^ | 5024; 4170–10820^c^ | 2294; 1193–3887 |

^a^Significant difference (*P* < 0.05) when compared to DCM patients; ^b^Significant difference (*P* < 0.05) when compared to DCM and MMVD patients; ^c^Significant difference (*P* < 0.05) when compared to MMVD patients.

**Table S3** Oxidative stress markers (mean ± SD) in DCM and MMVD patients and control dogs

|  | Control  n = 10 | DCM  n = 16 | MMVD  n = 2u1 |
| --- | --- | --- | --- |
| MDA (µmol/L) | 1.36 ± 0.31 | 1.71 ± 0.66 | 1.26 ± 0.77 |
| GPX (U/g Hgb) | 393.7 ± 43.6 | 403.0 ± 57.7 | 464.8 ± 48.9* |

* Significant difference (*P* < 0.05) when compared to control dogs and DCM patients
